# Ribosome biogenesis disruption mediated chromatin structure changes revealed by SRAtac, a customizable end to end analysis pipeline for ATAC-seq

**DOI:** 10.1186/s12864-023-09576-y

**Published:** 2023-09-01

**Authors:** Trevor F. Freeman, Qiuxia Zhao, Agustian Surya, Reed Rothe, Elif Sarinay Cenik

**Affiliations:** Department of Molecular Biosciences, University of Texas, Austin, TX 78712 USA

**Keywords:** Nucleolus, ATAC-seq, Chromatin accessibility, C. elegans, RNA polymerase I, ATAC-seq analysis pipeline

## Abstract

The nucleolus is a large nuclear body that serves as the primary site for ribosome biogenesis. Recent studies have suggested that it also plays an important role in organizing chromatin architecture. However, to establish a causal relationship between nucleolar ribosome assembly and chromatin architecture, genetic tools are required to disrupt nucleolar ribosome biogenesis. In this study, we used ATAC-seq to investigate changes in chromatin accessibility upon specific depletion of two ribosome biogenesis components, RPOA-2 and GRWD-1, in the model organism *Caenorhabditis elegans*. To facilitate the analysis of ATAC-seq data, we introduced two tools: SRAlign, an extensible NGS data processing workflow, and SRAtac, a customizable end-to-end ATAC-seq analysis pipeline. Our results revealed highly comparable changes in chromatin accessibility following both RPOA-2 and GRWD-1 perturbations. However, we observed a weak correlation between changes in chromatin accessibility and gene expression. While our findings corroborate the idea of *a feedback mechanism between ribosomal RNA synthesis, nucleolar ribosome large subunit biogenesis, and chromatin structure during the L1 stage of C. elegans development, they also prompt questions regarding the functional impact of these alterations on gene expression.*

**Supplementary Information:**

The online version contains supplementary material available at 10.1186/s12864-023-09576-y.

## Introduction

 While many general next-generation sequencing (NGS) analysis pipelines have been developed to simplify and increase the reproducibility of data analysis, there is still a need for fully customizable end-to-end pipelines that can cater to specific data types. One widely used NGS analysis technique is ATAC-seq, which identifies regions of high chromatin accessibility across the genome and enables differential analysis of chromatin structure between groups [1, 2]. However, current NGS analysis pipelines for ATAC-seq [3–6] have limitations, such as not including useful quality control (QC) steps for common model organisms, being restricted to a narrow set of reference genomes, not providing end-to-end analysis from raw reads to usable data, and not allowing for customization to keep up with new data analysis practices.

Here, we present SRAlign and SRAtac, two NGS data processing pipelines for ATAC-seq analysis that are reproducible, configurable, and extensible. Built on the Nextflow platform [7], SRAtac allows for end-to-end analysis of ATAC-seq data, including QC metrics, read filtering, peak calling, merging, and quantification. The pipelines are designed to be organism agnostic, compatible with any reference genome, and provide the option to run end-to-end from raw reads to peak counts or to perform downstream analysis steps on aligned data. Additionally, the pipelines have an extensible design which allows for the incorporation of new analyses or default parameters to stay current with emerging best practices in ATAC-seq analysis.

To better analyze ATAC-Seq data from *Caenorhabditis elegans*, a commonly used model organism for genome-wide ATAC-seq and ChIP-seq experiments, SRAtac offers unique functionalities not found in existing approaches. These functionalities include accounting for bacterial contamination, which is important since *C. elegans* is typically grown on a lawn of *E. coli*.

In this study, we used SRAtac to explore the relationship between chromatin structure and ribosomal RNA transcription in *C. elegans*. The relationship between nuclear chromatin and ribosomal RNA (rRNA) transcription is an essential aspect of cellular function, as it is directly related to the regulation of gene expression and protein synthesis. rRNA transcription occurs in the nucleolus, a specialized subnuclear compartment where early ribosome biogenesis takes place. The nucleolus forms around tandemly repeated ribosomal DNA (rDNA) sequences, which are transcribed by RNA polymerase I (Pol I) to produce rRNA(reviewed in [8]).

There is increasing evidence suggesting that rRNA transcription and ribosome biogenesis can impact global chromatin structure and dynamics. For instance, changes in ribosomal DNA (rDNA) copy numbers, which can affect nucleolus size, have been linked to the loss of heterochromatin-induced gene silencing throughout the genome [9, 10]. This implies that rDNA might have an epigenetic role in regulating gene expression. In addition, in metazoan organisms, the nucleolus is known to anchor specific regions of heterochromatin, called Nucleolar Associated Domains (NADs) [11–14]. However, it is still unclear whether the maintenance of NAD-associated heterochromatin is dependent on an intact nucleolus structure or active ribosome biogenesis. This is further complicated by the fact that the nucleolus also plays a role in ribosome synthesis, making it challenging to differentiate the effects of nucleolar structure disruption from those caused by protein synthesis.

To address these potential mechanisms, we depleted the RNA polymerase I component, RPOA-2, and a nucleolar ribosome biogenesis factor, GRWD-1, in embryogenesis and the first larval stage of post-embryonic development in *C. elegans* [15]. We hypothesized that disruptions in ribosomal RNA transcription or nucleolar large subunit assembly could alter chromatin structure and result in further changes in gene expression. By studying this relationship, we aim to shed light on the mechanisms underlying gene expression regulation during post-embryonic development. Additionally, understanding the feedback loop between chromatin and ribosome biogenesis in *C. elegans* could have broader implications for our understanding of how cells regulate gene expression and adapt to changes in their environment.

Our findings demonstrate that the depletion of proteins involved in ribosomal RNA transcription and nucleolar ribosome large subunit biogenesis leads to similar changes in chromatin accessibility during the L1 stage of post-embryonic development. We specifically observed comparable changes in chromatin accessibility upon the specific depletion of RPOA-2, and GRWD-1 in *C. elegans* [15]. These results indicate that nucleolar ribosome biogenesis has a crucial impact on the formation of chromatin structure during this developmental stage.

It is known that the nucleolus is an essential organelle that plays a critical role in ribosome biogenesis, which is necessary to produce functional ribosomes and the regulation of protein synthesis. Our results suggest that nucleolar ribosome synthesis also has a significant impact on the structure of chromatin, which can play a crucial role in shaping the gene expression program of cells. This is particularly important during post-embryonic development, as the organism is transitioning from a growth quiescence capable stage to adulthood with fully differentiated somatic and germline tissues. Our results highlight the importance of studying the relationship between ribosome biogenesis and chromatin structure to gain a deeper understanding of the regulation of gene expression.

## Results

### Implementation of SRAlign and SRAtac

SRAlign and SRAtac are two pipelines for NGS data processing that are built on the Nextflow platform. Nextflow is a workflow management software that simplifies the development of portable and reproducible bioinformatics workflows [7]. These pipelines were designed to be reproducible, configurable, and extensible, making them fully customizable end-to-end pipelines for specific data types.

SRAlign consists of a central processing workflow that carries out a range of NGS pre-processing steps, including read quality control, sequence alignments, library complexity, and sample reproducibility (Figure S1). This workflow currently supports two short read aligners, Bowtie2 and HISAT2 [7, 16]. SRAlign was designed to be extensible, allowing experienced users to add new modules to expand the workflow or build entirely new workflows on top of it. The workflow's parameters can be fully modified through Nextflow configuration files, YAML or JSON parameter files, or command line options.

**Fig. 1:**
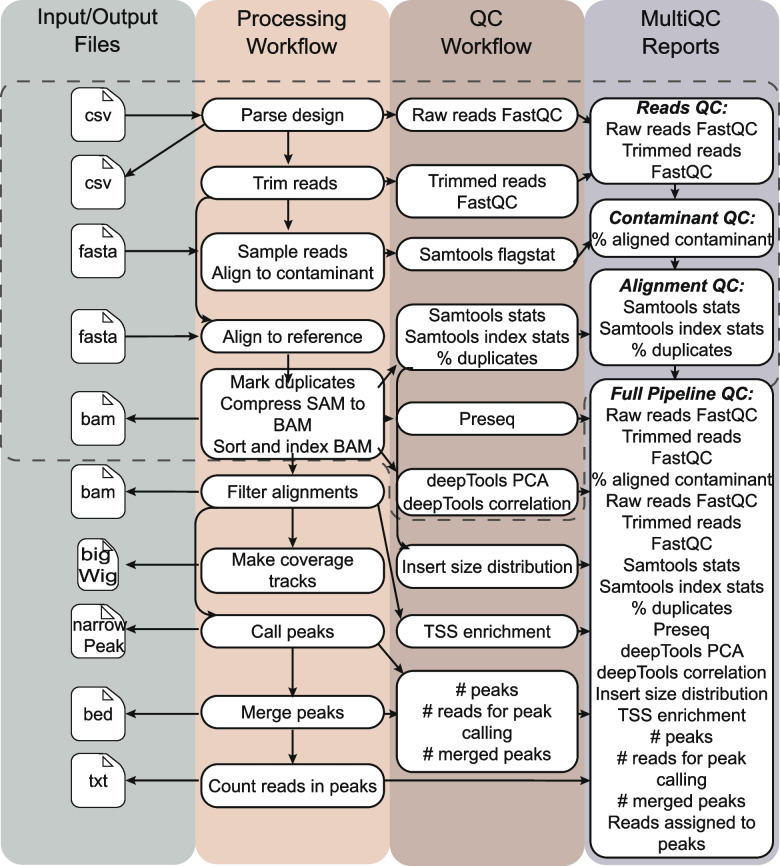
SRAtac extends SRAlign with ATAC-seq specific processing and quality control (QC) workflow steps. SRAlign steps are outlined with a dashed line. ATAC-seq specific processing and QC steps extend the SRAlign pipeline for analysis of ATAC-seq samples

SRAtac is an extension of SRAlign specifically designed for ATAC-seq data analysis [1, 2]. SRAtac comprises integrated processing and QC workflows that perform important steps in ATAC-seq data analysis, including filtering uninformative reads, generating coverage tracks in bigWig format, peak calling with MACS2 [17], peak merging, and read quantification for each peak (Fig. 1) [6]. The pipeline includes sensible default parameters for all ATAC-seq analysis steps, such as using the Nextera transposase insertion sequence as the default adapter sequence for trimming and a fragment length cutoff of 2,000 bases for sequence alignment using Bowtie2.

Processing steps include filtering uninformative reads (unmapped reads, reads mapping to mitochondrial chromosome, and those with low mapping quality), generating coverage tracks in bigWig format, peak calling with MACS2 [17], peak merging, and read quantification for each peak. The SRAtac QC workflow reports metrics including insert size distribution with transcription start site (TSS) enrichment. Similar to SRAlign, these QC metrics are written to a final MultiQC report for easy and interactive review [18]. Similar to SRAlign, SRAtac was designed to be extensible, allowing users to add new modules to expand the pipeline or build entirely new pipelines on top of it.

Both SRAlign and SRAtac are available as public repositories on GitHub, (https://github.com/trev-f/SRAlign,https://github.com/trev-f/SRAtac), allowing users to access the code, make modifications, and contribute to the development of these pipelines. Overall, these pipelines provide fully customizable and reproducible NGS data processing pipelines for specific data types, making them valuable tools for researchers in the field of genomics.

### SRAtac recapitulates key features of ATAC-seq data

We evaluated the performance of SRAlign and SRAtac by using publicly available RNA-seq and ATAC-seq datasets from *C. elegans*. First, we used two capped nuclear RNA-seq datasets from wild-type *C. elegans* embryos (GSM3142773 and GSM3142774, [19]) and processed them using SRAlign with default parameters and HISAT2 as the aligner to the ce10 reference genome. Both datasets were sampled to 200,000 total reads to test the software, and the resulting alignment rates were high with low *E. coli* contamination (Figure S2A). Our analysis of the distribution of reads mapped to each chromosome showed that the reads were biased towards aligning to chromosome I with the expected distribution within genes (Figure S2B, C).

**Fig. 2:**
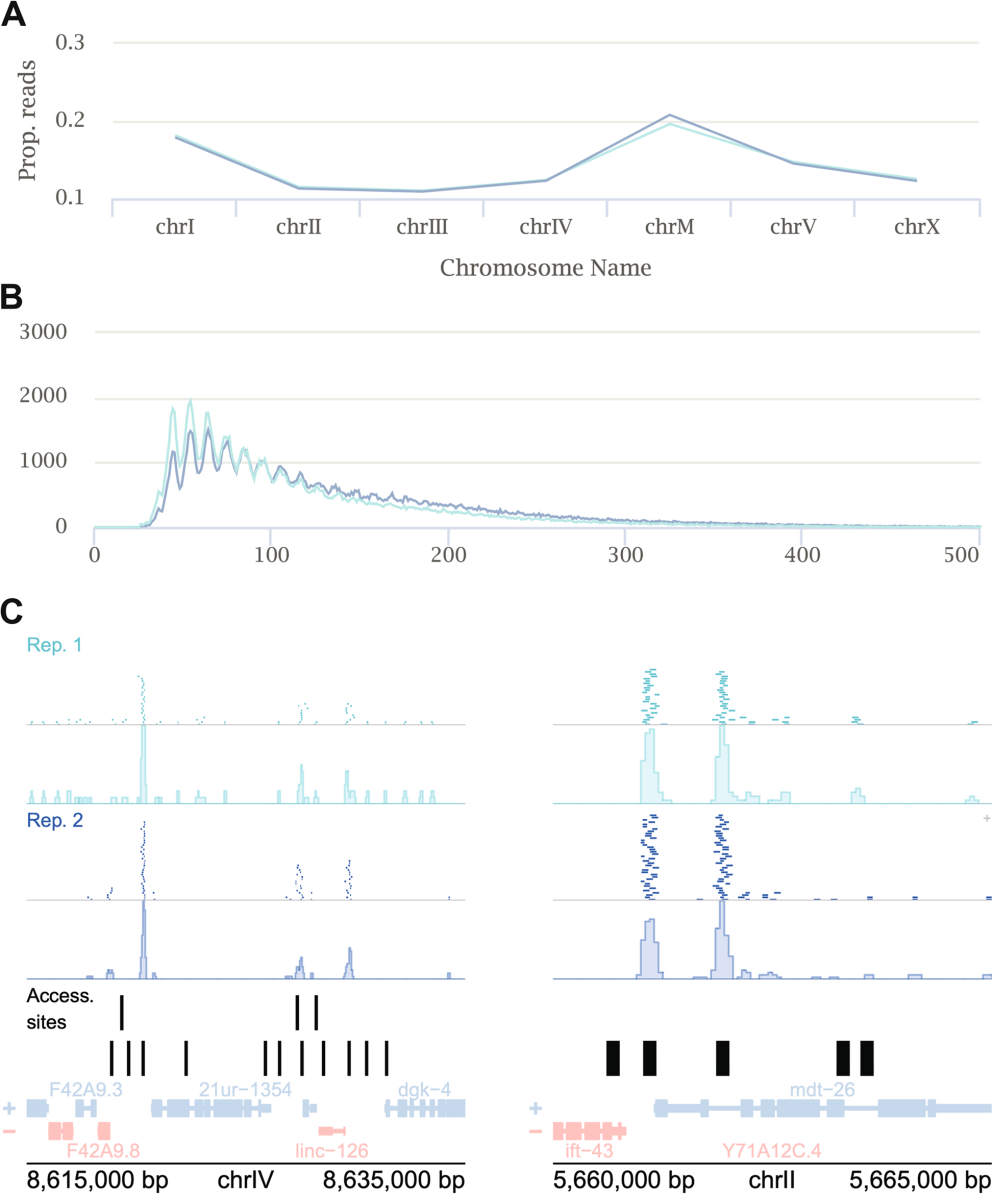
SRAtac captures key features of ATAC-seq data. **A** Proportion of aligned reads aligned to each chromosome. The light and dark blue color indicates two different replicates of ATAC-seq from Janes et al. 2018 [19]. chrM indicates mitochondrial DNA. **B** Insert size distribution of aligned ATAC-seq reads. **C** Genome browser snapshots of ATAC-seq alignments and bigWig coverage tracks for both replicates with published accessibility sites [19] and RefSeq genes

Furthermore, we evaluated SRAtac using previously published ATAC-seq datasets from hmg-3 RNAi-treated wild-type L4 stage *C. elegans* (GSM2715417 and GSM2715418, [20]). Our analysis revealed an overrepresentation of reads on chromosome I, which is consistent with the abundance of reads that align to the rDNA locus (Fig. 2A). Additionally, we observed an overrepresentation of reads mapping to mitochondrial DNA (chrM), which is a common feature of ATAC-seq datasets due to the mitochondrial DNA being more efficiently tagmented than the nuclear chromosomes as it is not packaged into chromatin [21]. Insert length distribution and example genome browser snapshots of detected peak regions in comparison to aligned reads are also shown (Fig. 2B,C). These results demonstrate the suitability and reliability of SRAlign and SRAtac for processing and analyzing RNA-seq and ATAC-seq data from *C. elegans*.

Our evaluation of publicly available datasets demonstrates that SRAtac accurately captures important characteristics of ATAC-seq data. Notably, these results also indicate that SRAlign is not limited to processing RNA-seq data, as it performs equally well on DNA-seq data. Therefore, SRAlign is a versatile and adaptable tool for short read NGS alignment, capable of serving as the foundation for more specialized pipelines.

### Ribosomal RNA transcription and Nucleolar Large Subunit ribosome biogenesis regulate chromatin accessibility at L1 stage of post-embryonic development in *caenorhabditis elegans*

**Fig. 3:**
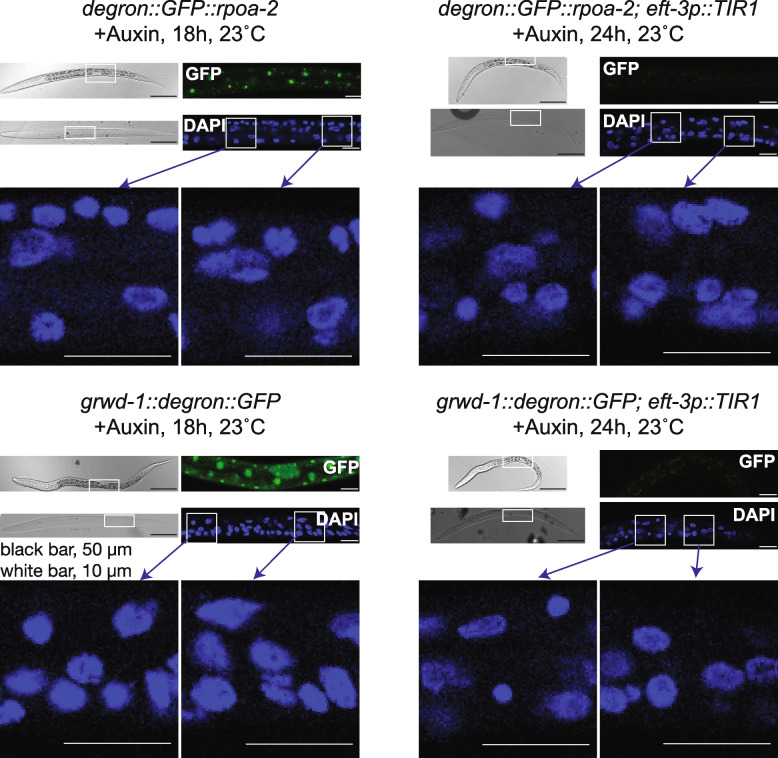
Depletion of RPOA-2 or GRWD-1 doesn’t result in major observable nuclear morphology differences at L1 stage. Depletion of RPOA-2 or GRWD-1 were confirmed in the presence of auxin, with or without globally expressed TIR1. *degron::GFP::rpoa-2* and *grwd-1::degron::GFP* strains have expected GFP localization patterns (GFP images on the left). Upon introduction of globally expressed TIR1 (*eft-3p::TIR1*), both RPOA-2 and GRWD-1 are depleted within 24 h in the presence of auxin (GFP images on the right). We did not observe significant overall morphology changes in the nucleus (DAPI staining images, no depletion on the left, with depletion on the right, RPOA-2: top section, GRWD-1: bottom section)

To investigate the chromatin level consequences of nucleolar perturbations, we employed an auxin inducible degradation (AID) system to specifically target RNA polymerase I and nucleolar large ribosome assembly at L1 stage in *C. elegans*. The AID system utilizes an AID degron cassette integrated into the genomic loci of a coding region of interest, where its protein product can be depleted upon expression of an auxin receptor F-box protein TIR1 and in the presence of auxin (IAA, Indole-3-acetic acid) [22, 23]. We integrated a degron-GFP cassette into the N-terminus of the second largest subunit of RNA polymerase I (*rpoa-2*) and the C-terminus of a chaperone protein coding gene (*grwd-1/Y54H5A.1*) required for nucleolar export and assembly of RPL-3 to the large ribosomal subunit [15]. The resulting *C. elegans* strains expressing degron-GFP integrated RPOA-2 and GRWD-1 exhibited homozygous viability with nucleolar RPOA-2 and nuclear GRWD-1 localization patterns (Fig. 3, left, GFP images [15]). The presence of auxin (IAA) depleted the full-length Degron::GFP::RPOA-2 protein and GRWD-1::Degron::GFP when TIR1 is expressed (Figure S3A [15]). Furthermore, upon RPOA-2 depletion, we observed a significant decrease in the abundance of internal transcribed spacer expression relative to mature 26S levels (Figure S3B). Upon auxin treatment of the degron-integrated *rpoa-2* or *grwd-1* embryos, the animals successfully go through embryogenesis due to sufficient loading of maternal ribosomes [24] but become arrested in growth during further post-embryogenesis [15]. At this stage, animals have abundant levels of maternal ribosomes, and zygotic ribosome production is required for further development post-embryogenesis.

During embryogenesis, nucleolar assembly dynamics are altered without ribosomal RNA transcription, but passive phase separation of nucleolar components still occurs [25, 26]. Consistent with this observation, neither depletion of RPOA-2 nor GRWD-1 leads to any significant observable changes in nuclear shape (Fig. 3, DAPI images, no depletion on the left and with depletion on the right). Additionally, post-embryonic growth arrest likely shields the general nuclear structure from crowding-mediated effects of a lack of new ribosomal RNA production.

To assess whether nucleolar ribosome biogenesis affects chromatin structure, we used ATAC-seq to measure chromatin accessibility changes upon depletion of RPOA-2 and GRWD-1. To define differentially accessible regions (DARs) upon RPOA-2 and GRWD-1 depletion, counts of reads within peaks were produced by SRAtac v0.5.0, which were then separately run through the DESeq2 workflow with batch effects controlled for by RUVseq [27].

**Fig. 4:**
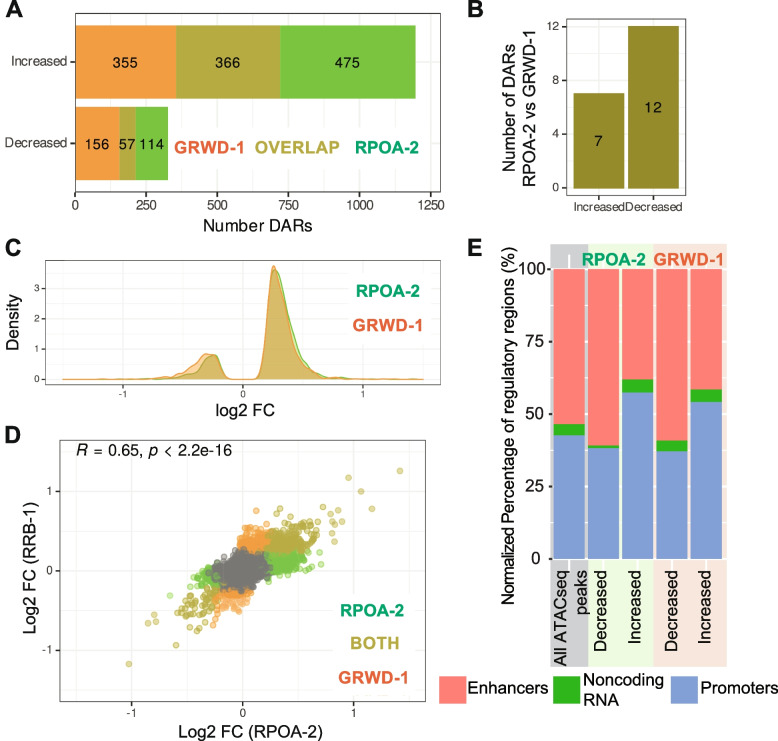
Degradation of RPOA-2 and GRWD-1 induce similar chromatin accessibility changes. **A** Numbers of differentially accessible regions (DARs) unique to or overlapped between RPOA-2 (green) and GRWD-1 (orange) depletion. **B** Number of DARs with increased or decreased accessibility specific to only RPOA-2 (green) or GRWD-1 (orange) depletion. **C** Density plots of shrunken log2 fold changes from DESeq2 results for all DARs for RPOA-2 (green) and GRWD-1 (orange) depletion. **D** Scatter plot of shrunken log twofold changes from DESeq2 results in RPOA-2 (green)and GRWD-1 (orange) depletion. P value from Pearson correlation test is shown (*R* = 0.65, *p* < 2.2e-16). **E** DARs were assigned to previously annotated *C. elegans* regulatory regions [19]; red: putative enhancers, green: noncoding RNA, blue: promoters. Percentages were normalized to genomic regulatory regions that overlap with all ATACseq peaks, or DARs

After removal of unwanted variations likely due to batch effects, principal component analysis showed that samples were primarily divided into two groups: those with a depleted ribosome biogenesis component and those without (Figure S4A, B). Differential accessibility analysis revealed that the majority of DARs have increased rather than decreased accessibility after depletion of either RPOA-2 or GRWD-1 (Fig. 4A and Figure S4C, D).

Depletion of RPOA-2 and GRWD-1 had broadly similar effects on chromatin accessibility. There is a large overlap of DARs with both increased and decreased accessibility, although there are a few distinct DARs within each of the two groups (Increased DARs: p ~ 0, Decreased DARs: *p* < 1e-81; hypergeometric test) (Fig. 4A). Differential accessibility analysis revealed only minor differences when RPOA-2 depleted larvae were compared to GRWD-1 depleted larvae (Fig. 4B). The distribution of log2 fold changes for DARs in both categories demonstrated a strong overlap (Fig. 4C). Log2 fold changes of DARs were also moderately correlated (R = 0.65, *p* < 2.2e-16, Pearson correlation) (Fig. 4D). Clustering of top DARs with increased and decreased accessibility for RPOA-2 or GRWD-1 degron further demonstrated the similarity between the depletion of these two ribosome biogenesis factors (Figure S4E, F). Altogether, this suggests that there is some feedback between chromatin structure and production of protein synthesis machinery in *C. elegans* as disrupting ribosome biogenesis in two ways led to similar changes in chromatin structure.

To understand what type of genomic regions were affected at the chromatin level upon GRWD-1 and RPOA-2 depletion, ATAC-seq peaks were merged with a list of experimentally determined *C. elegans* regulatory elements [10]. Significantly more accessible DARs upon RPOA-2 and GRWD-1 depletion are slightly biased towards being enriched among promoter regions in comparison to putative enhancers (Fig. 4E).

**Fig. 5:**
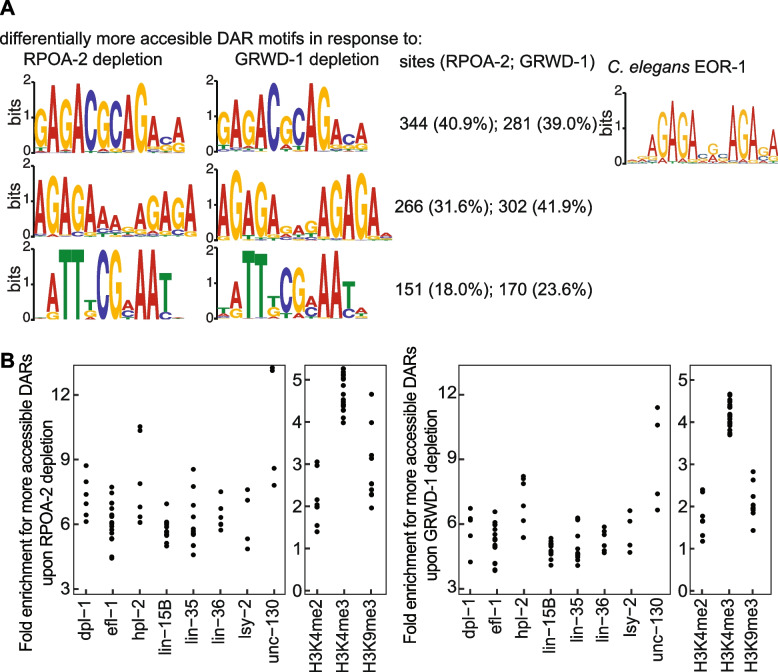
Motifs and transcription factor binding sites with significant enrichment within more accessible regions upon RPOA-2 and GRWD-1 depletion. **A** Left: Comparative motif analyses (STREME) of differentially accessible regions reveal similar motifs that are significantly enriched among regions that become more accessible following RPOA-2 and GRWD-1 depletion. Middle: The numbers of regions becoming significantly more accessible upon RPOA-2 and GRWD-1 depletion, which also show enrichment for the corresponding motif, are shown along with their respective percentages. Right: Close examination of the significant motifs revealed similarities to the EOR-1 binding site, which was downloaded from the JASPAR database [28], with colors adjusted accordingly. **B** We assessed significant enrichment across transcription factor binding sites or histone modification sites using data from ChipAtlas [29, 30]. This data was filtered based on significance (Q value < -2), specificity to the L1 stage, and inclusion in at least four different experiments. The y-axis displays relative enrichment, the x-axis shows different transcription factors and histone modifications, and each point represents a single experiment

We questioned whether DARs were enriched in any motif sequences. We discovered that the increased accessibility of regions upon RPOA-2 and GRWD-1 depletion corresponded with a significant enrichment of 3 similar motifs (Fig. 5A, left, Streme analysis, E-value < 0.05 [19]). In contrast, no significant motif was detected in the regions where accessibility significantly decreased.

As significantly more accessible DARs upon RPOA-2 and GRWD-1 depletion are slightly biased towards promoters and are enriched for multiple motif sequences, we searched for similar motif sequences among known transcription factor binding sites. Among regions with increased accessibility, a distinct motif, GAGACGCAGASA, is notable for its significant resemblance to the transcription factor EOR-1 (Fig. 5A, right). This motif's similarity corresponds with EOR-1 transcription factor-bound regions as observed in ChipAtlas [29]. Notably, approximately 57% (210 out of 366) of DARs common to both RPOA-2 and GRWD-1 depletions intersect with specific EOR-1 transcription factor-bound regions (*p*-value < 0.01, odds ratio = 22, Fisher’s Exact test). In contrast, a mere 6% of all ATACseq peaks coincide with EOR-1 bound regions [31]. Consequently, these findings indicate a specific enrichment of the EOR-1 binding motif and regions among DARs post-depletion of RPOA-2 and GRWD-1. EOR-1 is known to: (1) regulate RMED/V neuron specification in a Ras-independent manner [32], (2) drive P12 and vulval cell fate specification in a Hox-dependent way [33, 34], and (3) interact genetically with the SWI/SNF complex, affecting the expression of genes vital for hermaphrodite-specific HSN neuron maturation [35]. These observations emphasize that changes in chromatin structure following RPOA-2 and GRWD-1 depletion may influence the accessibility of genomic regions enriched for specific transcription factors pivotal for cell specification.

We further investigated whether other transcription factors or histone modifications were significantly enriched among DARs following the depletion of RPOA-2 and GRWD-1, using the ChipAtlas database [29]. We focused on significant enrichment of RPOA-2 GRWD-1 DARs with at least 4 different previously published Chip Seq datasets for each transcription factor or histone modification. As a result, we found additional transcription factors and chromatin binding proteins; DPL-1, EFL-1, HPL-2, LIN-15B, LIN-35, LIN-36, ISY-2, UNC-130, as well as histone modifications, H3K4me2, H3K4me3 and H3K9me3 were all significantly enriched among increased accessible regions upon RPOA-2 and GRWD-1 depletion (Fig. 5B).

Among the histone modifications, the most prominent one was H3K4 methylation. H3K4 methylation status has previously been reported to be associated with genome stability and DNA damage [36, 37]. It is known to be enriched along the bodies of coding genes, and it plays a regulatory role in activating ribosome biogenesis and protein homeostasis following recovery from UV induced DNA damage [38]. In light of this, we decided to compare the genes enriched with H3K4me2 (over two-fold enrichment in comparison to the H3 control) both with and without exposure to UV radiation 38]. We identified 571 genes associated with regions that are differentially more accessible upon both RPOA-2 and GRWD-1 depletion. Of these, 190 were associated with regions enriched with H3K4me2 following UV exposure (Fisher’s exact test, p-value < 0.01). Notably, no overlapping genes were found in H3K4me2 enriched regions when there was no exposure to UV radiation. Consistent with ATAC-seq, RNA-seq differences in response to RPOA-2 depletion reflects this similarity. Gene expression differences between UV-irradiated and RPOA-2 depleted larvae are notably similar; both overexpressed (odds ratio = 7.8, p-value < 2.2e-16) and underexpressed categories (odds ratio = 6.7, p-value < 2.2e-16) show significant overlap [15]. Collectively, these results suggest that regions becoming differentially more accessible after GRWD-1 and RPOA-2 depletion significantly coincide with regions enriched in H3K4me2 upon UV exposure. Moreover, they exhibit a gene expression response analogous to UV irradiation.

### Low Correlation between RPOA-2 dependent chromatin accessibility changes and gene expression changes

In order to investigate whether chromatin changes induced by disruption of nucleolar ribosome biogenesis result in altered cytoplasmic gene expression, RNA-seq was performed in the presence of auxin in L1 larvae in the *rpoa-2::degron* background with globally expressed TIR1 or absent altogether. DESeq2 analysis was performed on RNA-seq counts of reads within genes to identify differentially expressed genes (DEGs). To directly compare the ATAC-seq dataset with the RNA-seq dataset, ATAC-seq peaks were paired with their associated genes through *C. elegans* regulatory elements (Fig. 4E, [19]).

**Fig. 6:**
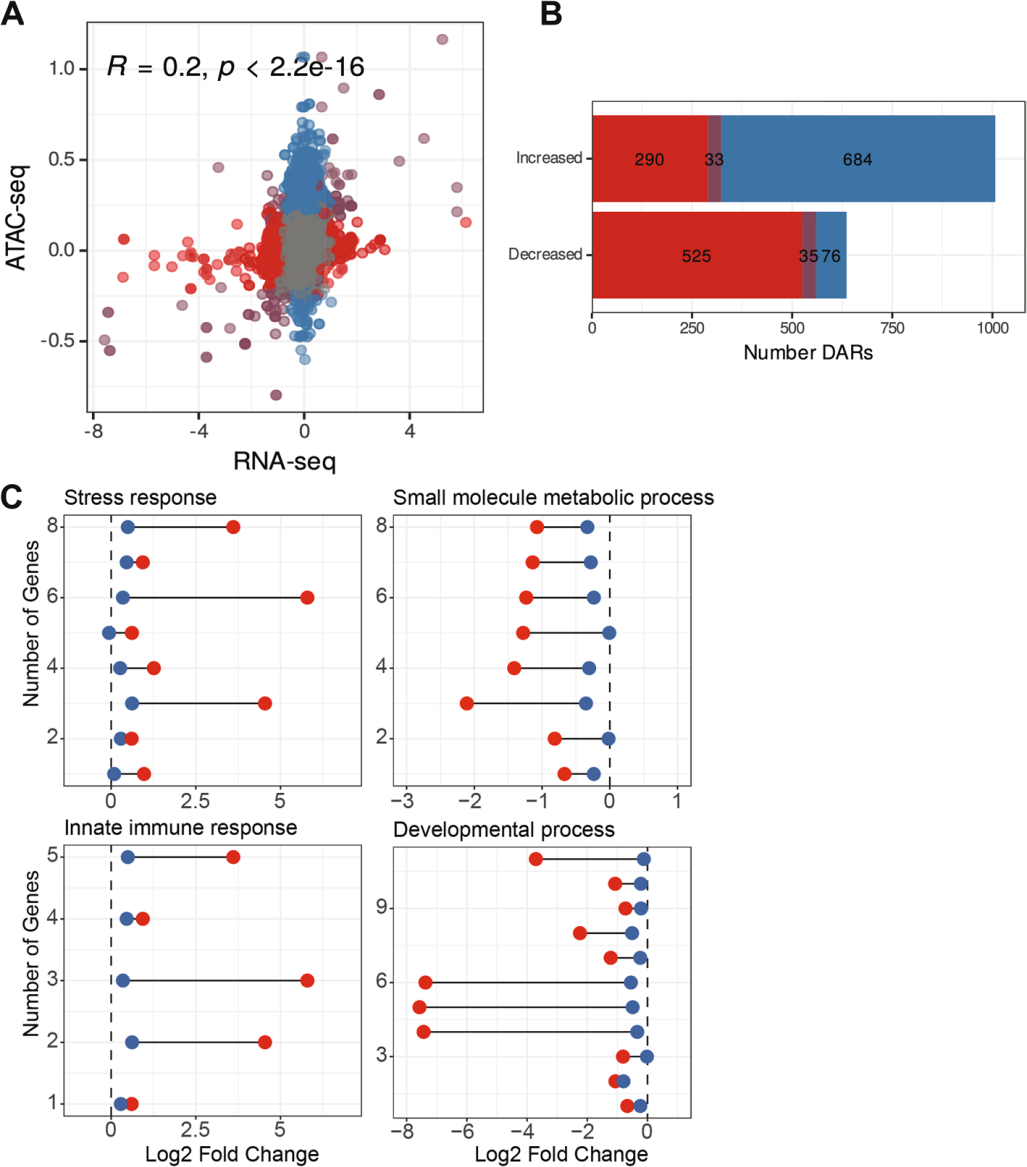
Correlation between chromatin accessibility and gene expression changes in response to RPOA-2 depletion. **A** Scatter plot of shrunken log twofold changes from DESeq2 results of ATAC-seq vs RNA-seq in response to RPOA-2 depletion. **B** Numbers of differentially accessible regions (DARs, blue), differentially expressed genes (DEGs, red), and overlap of ATAC-seq and RNA-seq (purple) in response to RPOA-2 depletion. **C** ATAC-seq (blue) and RNA-seq (red) log2 fold change in response to RPOA-2 depletion within significantly enriched GO attributes from FuncAssociate 3.0 [39]

The correlation between the log2 fold change of ATAC-seq peaks and their associated cytoplasmic gene expression is low but significant (R = 0.2, *p* < 2.2e-16) (Fig. 6A). Similarly, the overlap of ATAC-seq DARs and RNA-seq differentially expressed genes (DEGs) are significant but small in numbers Fig. 6B, S5 (33 genes in increased DARs and DEGs: *p* = 4.6e-6, 35 genes in decreased DARs and DEGs: *p* = 3.3e-25; hypergeometric test). The presence of a low correlation is not surprising as (1) most of the open regions likely require the presence of a transcription factor or enhancer for active transcription, and (2) posttranscriptional processes such as splicing, and RNA stability likely also affect mature RNA transcript levels in the cytoplasm. Given these caveats, significant but low correlation between ATAC-seq and RNA-seq suggests the presence of a relationship between nucleolar ribosome biogenesis and chromatin, which has an impact on overall gene expression in *C. elegans* L1 larvae.

To investigate the potential functions of the shared DARs and DEGs, FuncAssociate was used to determine significantly enriched Gene Ontology (GO) terms [39]. Among the 33 genes that become more accessible at chromatin level and are overexpressed upon RPOA-2 depletion, there was significant enrichment in stress and innate immune response (8 and 5 genes respectively, Fig. 6C, left, Table 1). Among the 35 genes that become less accessible at the chromatin level and are under expressed upon RPOA-2 depletion, a significant enrichment was observed in structure of cuticle, small molecule metabolic process and development (10, 8, and 11 genes respectively) (Fig. 6C, right, Table 2). Here, within these enriched GO categories, modest but significant changes in chromatin accessibility correlated with usually larger changes in transcript expression (Fig. 6C).

**Table 1:** FuncAssociate results for gene features associated with significantly increased accessibility DARs (ATAC-seq) and significantly over expressed DEGs (RNA-seq)

Number of Genes in the dataset	Total number of genes in GO category	Log 10 odds Ratio	P adjusted	Attribute ID	Attribute Name
5	198	1.2	0.032	GO:0045087	Innate immune response
5	199	1.2	0.032	GO:0006955	Immune response
5	203	1.2	0.034	GO:0002376	Immune system process
8	690	0.89	0.036	GO:0006950	Response to stress

**Table 2:** FuncAssociate results for gene features associated with significantly decreased accessibility DARs (ATAC-seq) and significantly under expressed DEGs (RNA-seq)

Number of Genes in the dataset	Total number of genes in GO category	Log 10 odds Ratio	P adjusted	Attribute ID	Attribute Name
2	5	2.54	0.034	GO:0010888	Negative regulation of lipid storage
3	13	2.23	0.001	GO:0060102	Collagen and cituculin-based cuticle
4	26	2.02	0.001	GO:0042338	Cuticle development
4	41	1.80	0.001	GO:0005578	Proteinaceous extracellular matrix
4	42	1.79	0.001	GO:0040002	Collagen and cituculin-based cuticle
4	44	1.77	0.001	GO:0042335	Cuticle development
4	49	1.72	0.001	GO:0031012	Extracellular matrix
10	170	1.65	< 0.001	GO:0042302	Structural constituent of cuticle
8	172	1.50	< 0.001	GO:0005581	Collagen trimer
11	376	1.34	< 0.001	GO:0005198	Structural molecule activity
8	573	0.96	0.016	GO:0044281	Small molecule metabolic process
11	1293	0.77	0.026	GO:0044767	Single molecule metabolic process
11	1364	0.74	0.043	GO:0032502	Developmental process

While there is a low global correlation between ATAC-seq and RNA-seq datasets, the correlated genes had significant enrichment in functional categories suggesting the role of chromatin organization in regulating functions such as stress response, metabolic process, and development.

## Discussion

### SRAlign and SRAtac

SRAlign is a flexible and reliable next-generation sequencing data processing workflow that can efficiently process both RNA-seq and DNA-seq data. SRAtac, built on top of SRAlign, offers a unique set of features that are not available in other ATAC-seq pipelines. Firstly, SRAtac includes a built-in analysis of contaminants which is particularly useful for the *C. elegans* research community. Secondly, SRAtac is organism and reference genome agnostic, meaning that any custom reference genome can be integrated, which is an improvement over other pipelines such as AIAP [3] and the ENCODE pipeline [40] that have limited reference genome options and do not support *C. elegans*. Thirdly, SRAtac can run in either end-to-end mode (from raw reads to counts within peaks) or in post-alignment mode (from BAM files to counts within peaks), which provides additional flexibility for further analysis without requiring computationally expensive steps such as read trimming and alignments to be repeated. This modular architecture enhances the existing pipelines that require aligned files such as ataqv [41] or those with a modular structure such as AIAP [3] or PEPATAC [5]. Finally, SRAtac is customizable, utilizing the powerful and highly modular Nextflow DSL2 language. We anticipate that SRAtac will be able to cater to the needs of any ATAC-seq experiment while also being adaptable to integrate future developments in new best practices for ATAC-seq data analysis.

### Chromatin structure and gene expression changes during postembryonic development in *C. elegans* upon depletion of ribosomal RNA synthesis and ribosome large subunit biogenesis component

In this study, we have demonstrated that *C. elegans* has a feedback mechanism between ribosome biogenesis and chromatin structure. Disruptions in ribosomal RNA transcription and nucleolar large subunit assembly result in altered chromatin structure in L1 larvae. Selective depletion of either of the ribosome biogenesis factors – the RNA Pol I subunit RPOA-2 or GRWD-1, which is responsible for large subunit biogenesis – causes changes in chromatin accessibility at specific regions. Interestingly, most of these regions have increased accessibility, suggesting that heterochromatin is disrupted (Fig. 4A) This finding aligns with a Drosophila study in which increased gene expression was observed in typically silenced heterochromatic regions following ribosomal DNA loss [9, 10]. In contrast, a study on mouse embryonic stem cells (mESCs) revealed that the state of hyper-transcription in mESCs relies on translation output. Specifically, disrupting translation with the inhibitor cycloheximide led to a depletion of euchromatin and disrupted RNA Pol II-mediated transcription [42]. One possible explanation for these differences is that when ribosome biogenesis is hindered, the maternally deposited ribosomes remain functional.

Chromatin accessibility changes were highly similar overall between RPOA-2 depleted larvae and GRWD-1 depleted larvae, indicating that the mechanism by which these two ribosome biogenesis components alter global chromatin accessibility is likely similar at the L1 stage. These regions are notably enriched (~ 40%) within the binding areas of the transcription factor EOR-1. Serving as a positive regulator of Ras/Wnt signaling pathways, EOR-1 is instrumental in the lineage specification of neurons, vulval cells, and excretory cells [32–34]. These findings imply that these EOR-1-bound regions may not be universally accessible across all lineages but could exhibit changes in chromatin architecture in particular contexts. Significant evidence indicates that EOR-1 operates within open chromatin regions that are bound by the SWI/SNF complex [35]. Moreover, EOR-1 is implicated in the expression of stress-responsive genes independently of *skn-1* (the gene encoding the human NRF2 ortholog) [43]. Consequently, EOR-1 might be directly or indirectly involved in the increased accessibility and expression of stress-related genes following the depletion of ribosomal RNA transcription and the large subunit assembly factor.

Among histone modifications, we found significant overlap between regions that become differentially more accessible following GRWD-1 and RPOA-2 depletion and regions enriched in H3K4me2 following UV exposure [38]. Similarly, we observed considerable overlap between RNA-seq datasets from RPOA-2 depleted and UV-irradiated larvae [15, 44]. These findings suggest an intriguing possibility: that the depletion of ribosome biogenesis factors and UV exposure may trigger a set of shared pathways that preferentially open up H3K4me2 regions. Further research is required to elucidate the mechanisms behind these observations.

Chromatin accessibility changes were only weakly correlated with gene expression changes, suggesting that chromatin accessibility changes alone are not sufficient for transcription activation. Furthermore, it is likely that other factors, such as the availability of transcription factors or post-transcriptional processes, also contribute to gene expression changes. A limitation of this study is that it relies heavily on the results of one assay, ATAC-seq, which has limited power to answer certain questions due to technical and analytical constraints. For example, ATAC-seq only provides an indication of the overall openness or closedness of chromatin. Potentially informative techniques to further probe these questions include chromosome conformation capture derived NGS techniques to interrogate global genome contacts, and imaging techniques such as 3D ATAC-PALM and immunofluorescence imaging of chromatin marks to visualize changes in chromatin accessibility and spatial distributions within the nuclear volume.

Overall, our results suggest the existence of a feedback loop to chromatin upon disruption of ribosome biogenesis, specifically at the stages of ribosomal RNA transcription and nucleolar large subunit assembly during the L1 stage of post-embryonic development. Considering that the depletion of RPOA-2 influences the overall levels of ribosome synthesis, including 40S, it is reasonable to hypothesize that this mechanism extends to other aspects of nucleolar ribosome biogenesis. This feedback mechanism likely plays a role in regulating functions such as stress response, metabolic process, and development, as suggested by the enrichment analysis of differentially accessible genes [19, 42, 45, 46].

## Materials and methods

### Software described in this manuscript

SRAlign and SRAtac are available as a public repository hosted on GitHub (https://github.com/trev-f/SRAlign, https://github.com/trev-f/SRAtac).

Project name: e.g. My bioinformatics project.

Project home page: e.g. http://sourceforge.net/projects/mged

Operating system(s): e.g. Platform independent.

Programming language: e.g. Java.

Other requirements: e.g. Java 1.3.1 or higher, Tomcat 4.0 or higher.

License: e.g. GNU GPL, FreeBSD etc.

Any restrictions to use by non-academics: e.g. license needed.

### Availability of data and materials

ATAC-seq and RNA-seq libraries are available through NCBI GEO, with accession code GSE218280 and GSE213367, with these links: https://www.ncbi.nlm.nih.gov/geo/query/acc.cgi?acc=GSE213367, https://www.ncbi.nlm.nih.gov/geo/query/acc.cgi?acc=GSE218280, respectively.

### Strain maintenance

*C. elegans* strains were maintained and grown at 16 °C or 20 °C on peptone enriched NGM agar plates seeded with a lawn of *E. coli* OP50 bacteria. To obtain synchronized embryos, a standard bleaching procedure was used.

### Preparation of datasets for SRAlign and SRAtac testing

Datasets GSM3142773 and GSM3142774 for SRAlign and GSM2715417 and GSM2715418 for SRAtac were downloaded as raw reads fastq files from the Sequence Read Archive. Raw reads were sampled by processing both ends of the paired end reads files with the command line tool `seqtk sample` and option `-s100` to set the seed and preserve read pairs. For pipeline testing, datasets were sampled to 200,000 reads each for both SRAlign and SRAtac testing. These sampled datasets were exclusively used for this purpose.

### Data processing for SRAlign and SRAtac tests

RNA-seq reads were run through the SRAlign workflow version v1.0.2 to align reads to reference genome assembly ce10. The run was performed using the Docker profile with default parameters except `–alignmentTool hisat2 –skipPreseq` were used to perform the alignment of reads to the reference genome with HISAT2 and avoid errors with Preseq that can occur when alignment is performed with HISAT2. Visualizations for the dataset QC were saved directly from the final MultiQC report, and genome browser plots were produced using plotGardener [47].

ATAC-seq reads were run through the SRAtac workflow version v0.5.0 to align reads to reference genome assembly ce10. The run was performed using the Docker profile with all default parameters. Visualizations for the dataset QC were saved directly from the final MultiQC report, and genome browser plots were produced using plotGardener ^48^. Accessible sites were obtained from a published report compiled from accessible regions at numerous developmental and aging timepoints [19].

### Sample and library preparation for ATAC-seq

Bleach synchronized embryos were placed on NGM agar plates with a lawn of OP50 bacteria and supplemented with 1 mM auxin. Larvae were synchronized at the L1 stage by allowing the embryos to hatch and develop at 23 °C for 18 h for strains with no *tir-1* and 24 h for strains with *tir-1* as these have a growth delay. Synchronized larvae were collected in 50 mM NaCl. Bacteria were cleaned from worms by sedimentation through 5% sucrose in 50 mM NaCl at 400* g* for 1 min with subsequent washes in 50 mM NaCl. At least 40,000 larvae were collected for each condition. Batches of larvae were evenly split into four replicates and buffer was exchanged to Nuclei Wash Buffer (NWB): 10 mM Tris–HCl pH 7.5, 40 mM NaCl, 90 mM KCl, 3 mM MgCl_2_, 0.2 mM DTT, 0.5 mM Spermidine, 0.25 mM Spermine, and 1 X Protease Inhibitor Cocktail. Larvae pelleted in NWB were snap frozen in liquid nitrogen and stored at -70 °C.

In order to minimize the potential confounding effects of batch on the experimental design, one replicate of each of the strains was processed concurrently for four total batches of four replicates. To isolate nuclei from larvae, worm pellets were thawed on ice. Buffer was transferred for thawed larvae to Nuclei Prep Buffer (NPB): 10 mMTris-HCl pH 7.5, 40 mM NaCl, 90 mM KCl, 3 mM MgCl2, 0.2 mM DTT, 0.5 mM Spermidine, 0.25 mM Spermine, 0.1% Triton X-100, 0.1% IGEPAL CA-630, and 1 X Protease Inhibitor Cocktail. Worms were transferred to a 2 mL dounce homogenizer that was pre-chilled in the refrigerator and kept on ice. Worms were initially broken by 15 strokes with homogenizer pestle A, a short rest on ice, and 15 more strokes with pestle A. Nuclei were released from broken worms by 25 strokes with pestle B, a short rest on ice, and 25 more strokes with pestle B. Debris was pelleted by a low speed spin of the homogenate for 5 min at 100* g* at 4 °C. To pellet nuclei, the supernatant was taken from the low speed spin and sedimented for 10 min at 2000* g* at 4 °C. Meanwhile, more nuclei were released by a second round of 25 strokes with pestle B, a short rest on ice, and 25 more strokes with pestle B. Debris was pelleted as above, and nuclei were pelleted as above in the same tube as the previous nuclei pellet. Nuclei yields were further increased with light sonication. Debris was resuspended in 300 μL total NPB and sonicated in a Diagenode Bioruptor on Low power for 2 cycles of 30 s ON / 30 s OFF. Volume was brought to 1 mL total, and debris was removed, and nuclei were pelleted in the same tube as above. Nuclei were subjected to a buffer exchange and cleanup step to further remove any debris. The nuclei pellet was resuspended in 1 mL Tagmentation Reaction Buffer (TRB): 1 X Tagment DNA buffer (10% Dimethylformamide, 10 mM Tris–HCl pH 7.5, 10 mM MgCl_2_), 0.33 X PBS, 0.01% Digitonin, 0.1% Tween-20. Debris was pelleted as above, and the supernatant was moved to a new tube and nuclei were pelleted as above. Nuclei were cleaned a final time by resuspending in TRB and pelleting. The supernatant was carefully removed to leave about 50 – 60 μL of supernatant without disturbing the nuclei pellet.

Nuclei were stained with DAPI and counted using a hemocytometer. Nuclei were tagmented by taking an aliquot of 50,000 nuclei, bringing the total reaction volume to 47.5 μL with TRB, and adding 2.5 μL TDE1 tagmentase. The tagmentation reaction was incubated for 30 min at 37 °C with shaking. Immediately after the reaction concluded, reactions were cleaned up using the Zymo DNA Clean & Concentrator-5 kit.

Cleaned up tagmented DNA was added to a PCR reaction mix with 1.25 µM each Nextera i5 and i7 adapters and NEBNext High-fidelity 2X PCR Master Mix. Libraries were amplified with an initial extension and denaturation step of 5 min at 72 °C and 30 s at 98 °C followed by 12 cycles of 10 s at 98 °C, 30 s at 63 °C, and 1 min at 72 °C, with a final extension of 10 min at 72 °C. Adapter contamination was initially removed with the Zymo DNA Clean & Concentrator-5 kit, and library size distributions were checked by running on a gel. Libraries were subjected to a final cleanup with 1.8 X AMPure XP beads.

### Sample and library preparation for RNA-seq

RNAseq library preparation is discussed [15]. Larvae with or without auxin treatment were collected in 50 mM NaCl and were cleaned from OP-50 bacteria by sedimentation through a 5% sucrose cushion including 50 mM NaCl. After sucrose clean-up of bacteria, larvae were flash frozen in 20 mM Tris- HCl pH 7.4, 150 mM NaCl, 5 mM MgCl2 and ground in liquid nitrogen with mortar and pestle. The frozen worm powder was thawed on ice and mixed with 5 mM DTT, 1% Triton X-100, 100 µg/ml cycloheximide (Sigma Aldrich) and 5 U/ml Turbo DNase (Thermo Fisher Scientific). 1 ml TRIzol (Thermo Fisher Scientific) was added to the lysate, vortexed and incubated 5 min at room temperature. To extract RNA, 200 ml volume of chloroform was added, then the sample was mixed and spun at 15,000 rpm for 10 min. Aqueous layer was used for further RNA precipitation. Isolated RNA was isopropanol precipitated and 80% ethanol washed. Thermostable RNAseH (Lucigen) and a pool of 94 DNA oligonucleotides antisense to *C. elegans* ribosomal RNA were used to deplete rRNA from 100 ng total *C. elegans* RNA [48]. RNA-seq libraries were prepared using SMARTer Stranded RNA-Seq kit (Clontech). Initially, RNA was alkaline fragmented at 95˚C for 4 min followed by the protocol optimized < 10 ng RNA input. 12–14 cycles of PCR were used to amplify the sequences. Library DNA was then purified using Agencourt AMPure XP beads (Beckman Coulter). The resulting libraries were quantified with Qubit dsDNA HS Assay Kit (ThermoFisher Scientific) and sequenced on NovaSeq 6000 v1.5, SP flow cell (Illumina).

### ATAC-seq data processing

ATAC-seq raw reads were processed using the SRAtac pipeline version v0.5.0 with the Docker profile on the Google Cloud Life Sciences executor. Reads were aligned to *C. elegans* reference assembly WBcel235 and contamination levels checked against the *E. coli* genome assembly EB1. Reads were trimmed using fastp with the Nextera adapter sequence specified. Trimmed reads were aligned using bowtie 2 with parameters `–very-sensitive -X 2000`. Duplicate reads were marked with SAMBLASTER [49], and SAM files were sorted, indexed, and compressed using SAMtools [50]. BAM files were filtered using Sambamba view with parameters `–filter "mapping_quality >  = 10 and not ref_name =  = 'MtDNA' and not unmapped" –format bam –with-header` then sorted and indexed using Sambamba. Filtered BAM files were converted to BED files with bedTools bamtobed, and peaks were called on BED files using MACS2 with parameters `–format BED –gsize ce –keep-dup 1 –nolambda –nomodel –extsize 150 –shift 75`. All narrowPeaks files were merged using Homer mergePeaks with options `-d given -gsize ce`. featureCounts was used to count reads within merged peaks with the option `–ignoreDup` set to prevent counting duplicate reads.

### Differential accessibility analysis

The counts matrix of reads within peaks was read into R. The `ruvg` function from the R package RUVseq was used to remove unwanted variation that was likely due to batch effects ^52^. The set of empirical control peaks for `ruvg` were obtained by running DESeq2 on the counts matrix. Peaks with an adjusted p-value greater than or equal to 0.75 in the comparison *rpoa-2::degron; eft-3p::tir-1* vs. *rpoa-2::degron* were extracted as empirical control genes. Two factors of unwanted variation were computed for the data. Differential peaks were called using DESeq2 with the two factors of unwanted variation and the genotype as variables in the design ^28^. The R package apeglm was used for shrinkage of log fold changes [51].

### Whole genome sequencing library preparation

Illumina Nextera XT protocol was followed by using a suggested amount of genomic DNA. The resulting PCR product was cut from the gel at approximately 300 bp. The gel was eluted using Zymo gel purification kit and sequenced on the same Novaseq platform.

### Motif enrichment

Motifs were identified using the STREME software from MEME Suite (version 5.5.2) [52]. To extract sequences for motif analysis, coordinates for all open chromatin regions (OCRs) or differentially accessible regions (DARs) were exported from the `DESeqResults` object in R to text files in BED format using a custom script. Sequences were then extracted using `bedtools getfasta` (v2.31.0) to get the fasta sequence over each OCR or DAR from the WBCel235 reference genome. These sequences were used as input into STREME (v5.5.2) with the sequences of all OCRs provided as controls and DARs of either increased or decreased accessibility in either *rpoa-2::tir-1* vs. *rpoa-2* or *grwd-1::tir-1* vs. *grwd-1* as the sequences in which to find motifs. Transcription factor binding motifs were inspected and downloaded from JASPAR 2022 [28].

### Transcription factor and histone modification intersection analysis

EOR-1 transcription factor binding bed format data from L1 larvae was downloaded from ChipAtlas. The bedtools intersect was used with parameters -wa and -u to count the number of differentially accessible regions (DARs) that intersected. Fisher's exact test was used for DARs that intersect or do not intersect with EOR-1 binding sites.

For specific transcription factor and histone modification enrichments, Chip Atlas enrichment analysis tool was used [29]. Specifically, significantly more accessible DAR peaks upon RPOA-2 or GRWD-1 depletion were used as an input and all detected ATACseq peaks were used as a background. Significant enrichment data is reported only when it was detected at least 4 different set of Chip seq experiments for a given transcription factor or histone modification.

### Supplementary Information

**Additional file 1: Figure-S1. **SRAlign consists of a central processing workflow that carries out standard NGS processing steps, and writes key output files. Quality control (QC) workflow generates QC metrics for reads, alignments, library complexity, and sample reproducibility. Intermediate MultiQC reports and a final pipeline report includes output files as summarized. **Figure S2.** SRAlign effectively aligns RNA-seq data to a reference genome. A. Percentage of reads aligned [20]. B.Proportion of aligned reads aligned to e ach chromosome.C.Genome browser snapshots of RNA-seq alignments for both replicates with RefSeq genes.** Figure S3.** Validation experiments for RPOA-2 and GRWD-1 depletion. A. Depletion of GRWD-1::degron::GFP after 18 hours of auxin treatment. The numbers on top of the blot represent the ratio of GFP to actin. B. RT-qPCR showing the relative expression levels of the internal transcribed spacer of ribosomal RNA ITS1 and ITS2 in response to RPOA-2 depletion. The relative transcript levels were determined for each sample by normalizing them to mature 26S rRNA. Embryos of were treated with or without auxin for 18 hours. Data were obtained from three biological experiments with three technique replicates for each biological replicate. Statistical significance was determined using an independent t-test. **Figure S4.** Ribosome biogenesis disruption leads to significant changes in chromatin accessibility. A-B. PCA plots for the first principal components computed by DESeq2, prior to (A) and after (B) batch effect removal using RUVseq and limma::removeBatchEffect(). Green: no RPOA-2 depletion (RPOA-2); dark green: RPOA-2 depleted (RPOA-2; TIR1) light orange: no RRB-1 depletion (RRB-1); dark orange: RRB-1 depleted (RRB-1; TIR1) C-D. Number of differentially accessible regions (DARs) with increased–left– and decreased–right– accessibility for RPOA-2 (C) RRB-1 depletion (D). E-F. Heatmaps of log transformed normalized peak counts for the top 25 DARs in response to RPOA-2 (E) and RRB-1 (F) depletion. **Figure S5.** Most gene expression changes are not associated with chromatin accessibility changesVolcano plot of -log10 of adjusted p-value vs log2 fold change of ATAC-seq peaks overlapping annotated regulatory elements in response to RPOA-2 depletion. Points colored according to whether a particular peak is a differentially accessible region (DAR, green), or differentially expressed gene (DEG, purple), or both (navy).

## Data Availability

ATAC-seq and RNA-seq libraries are available through NCBI GEO, with accession code GSE218280 and GSE213367.
