# Supplementary material for: Ribosome biogenesis disruption mediated chromatin structure changes revealed by SRAtac, a customizable end to end analysis pipeline for ATAC-seq
Source: BMC Genomics. 2023 Sep 1;24:512. doi: 10.1186/s12864-023-09576-y (PMC10472662; doi:10.1186/s12864-023-09576-y)
Supplement: Supplementary file 1 — Additional file 1: Figure-S1. SRAlign consists of a central processing workflow that carries out standard NGS processing steps, and writes key output files. Quality control (QC) workflow generates QC metrics for reads, alignments, library complexity, and sample reproducibility. Intermediate MultiQC reports and a final pipeline report includes output files as summarized. Figure S2. SRAlign effectively aligns RNA-seq data to a reference genome. A. Percentage of reads aligned [20]. B.Proportion of aligned reads aligned to e ach chromosome.C.Genome browser snapshots of RNA-seq alignments for both replicates with RefSeq genes. Figure S3. Validation experiments for RPOA-2 and GRWD-1 depletion. A. Depletion of GRWD-1::degron::GFP after 18 hours of auxin treatment. The numbers on top of the blot represent the ratio of GFP to actin. B. RT-qPCR showing the relative expression levels of the internal transcribed spacer of ribosomal RNA ITS1 and ITS2 in response to RPOA-2 depletion. The relative transcript levels were determined for each sample by normalizing them to mature 26S rRNA. Embryos of were treated with or without auxin for 18 hours. Data were obtained from three biological experiments with three technique replicates for each biological replicate. Statistical significance was determined using an independent t-test. Figure S4. Ribosome biogenesis disruption leads to significant changes in chromatin accessibility. A-B. PCA plots for the first principal components computed by DESeq2, prior to (A) and after (B) batch effect removal using RUVseq and limma::removeBatchEffect(). Green: no RPOA-2 depletion (RPOA-2); dark green: RPOA-2 depleted (RPOA-2; TIR1) light orange: no RRB-1 depletion (RRB-1); dark orange: RRB-1 depleted (RRB-1; TIR1) C-D. Number of differentially accessible regions (DARs) with increased–left– and decreased–right– accessibility for RPOA-2 (C) RRB-1 depletion (D). E-F. Heatmaps of log transformed normalized peak counts for the top 25 DARs in response to RP [file 12864_2023_9576_MOESM1_ESM.zip › Freeman et al Supplementary Figure legends.docx]

**SUPPLEMENTARY FIGURE LEGENDS:**

**Figure S1:** **SRAlign automates data processing, performs quality control, and generates output files and MultiQC reports.** SRAlign consists of a central processing workflow that carries out standard NGS processing steps and writes key output files. Quality control (QC) workflow generates QC metrics for reads, alignments, library complexity, and sample reproducibility. Intermediate MultiQC reports and final pipeline report includes output files as summarized on the right side.

**Figure S2:** **SRAlign effectively aligns RNA-seq data to a reference genome.** **A.** Percentage of reads aligned [20]. **B.** Proportion of aligned reads aligned to each chromosome. **C.** Genome browser snapshots of RNA-seq alignments for both replicates with RefSeq genes.

**Figure S3: Validation experiments for RPOA-2 and GRWD-1 depletion**

**A.** Depletion of GRWD-1::degron::GFP after 18 hours of auxin treatment. The numbers on top of the blot represent the ratio of GFP to actin. **B.** RT-qPCR showing the relative expression levels of the internal transcribed spacer of ribosomal RNA ITS1 and ITS2 in response to RPOA-2 depletion. The relative transcript levels were determined for each sample by normalizing them to mature 26S rRNA. Embryos were treated with or without auxin for 18 hours. Data were obtained from three biological experiments with three technique replicates for each biological replicate. Statistical significance was determined using an independent t-test.

**Figure S4: Ribosome biogenesis disruption leads to significant changes in chromatin accessibility**. **A-B.** PCA plots for the first principal components computed by DESeq2, prior to (**A**) and after (**B**) batch effect removal using RUVseq and limma::removeBatchEffect(). Green: no RPOA-2 depletion (RPOA-2); dark green: RPOA-2 depleted (RPOA-2; TIR1); light orange: no GRWD-1 depletion (GRWD-1); dark orange: GRWD-1 depleted (GRWD-1; TIR1) **C-D.** Number of differentially accessible regions (DARs) with increased–left– and decreased–right– accessibility for RPOA-2 (**C**) GRWD-1 depletion (**D). E-F.** Heatmaps of log transformed normalized peak counts for the top 25 DARs in response to RPOA-2 (**E**) and GRWD-1 (**F**).

**Figure S5: Most gene expression changes are not associated with chromatin accessibility changes**. Volcano plot of -log10 of adjusted p-value vs log2 fold change of ATAC-seq peaks overlapping annotated regulatory elements in response to RPOA-2 depletion. Points colored according to whether a particular peak is a differentially accessible region (DAR, green), differentially expressed gene (DEG, purple), or both (navy).
